# An Educational Network for Surgical Education Supported by Gamification Elements: Protocol for a Randomized Controlled Trial

**DOI:** 10.2196/21273

**Published:** 2020-12-14

**Authors:** Natasha Guérard-Poirier, Michèle Beniey, Léamarie Meloche-Dumas, Florence Lebel-Guay, Bojana Misheva, Myriam Abbas, Malek Dhane, Myriam Elraheb, Adam Dubrowski, Erica Patocskai

**Affiliations:** 1 Faculty of Medicine Université de Montréal Montreal, QC Canada; 2 Department of General Surgery Université de Montréal Montreal, QC Canada; 3 Faculty of Health Sciences Ontario Tech University Oshawa, ON Canada; 4 Department of Surgical Oncology Centre Hospitalier de l’Université de Montréal Montreal, QC Canada

**Keywords:** distance learning, COVID-19, surgical pedagogy, learning platform, subcuticular sutures, advanced sutures, medical education, peer learning, surgery, medical student, web-based learning, web-based tool, gamification, video

## Abstract

**Background:**

Traditionally, medical students have learned surgical skills by observing a resident physician or surgeon who is performing the technique. Due to inconsistent practice opportunities in the clinical setting, a disparity of skill levels among students has been observed. In addition, the poor availability of faculty professors is a limiting factor in teaching and adequately preparing medical students for their clerkship years. With the ongoing COVID-19 pandemic, medical students do not have access to traditional suturing learning opportunities. Didactic courses are available on videoconferencing platforms; however, these courses do not include technical training.

**Objective:**

Our overarching goal is to evaluate the efficacy and usability of web-based peer-learning for advanced suturing techniques (ie, running subcuticular sutures). We will use the Gamified Educational Network (GEN), a newly developed web-based learning tool. We will assess students’ ability to identify and perform the correct technique. We will also assess the students’ satisfaction with regard to GEN.

**Methods:**

We will conduct a prospective randomized controlled trial with blinding of expert examiners. First-year medical students in the Faculty of Medicine of Université de Montréal will be randomized into four groups: (1) control, (2) self-learning, (3) peer-learning, and (4) peer-learning with expert feedback. Each arm will have 15 participants who will learn how to perform running subcuticular sutures through videos on GEN. For our primary outcome, the students’ ability to identify the correct technique will be evaluated before and after the intervention on GEN. The students will view eight videos and rate the surgical techniques using the Objective Structured Assessment of Technical Skills Global Rating Scale and the Subcuticular Suture Checklist as evaluation criteria. For our secondary outcomes, students will anonymously record themselves performing a running subcuticular suture and will be evaluated using the same scales. Then, a survey will be sent to assess the students’ acceptance of the intervention.

**Results:**

The study will be conducted in accordance with the Declaration of Helsinki and has been approved by our institutional review board (CERSES 20-068-D). No participants have been recruited yet.

**Conclusions:**

Peer learning through GEN has the potential to overcome significant limitations related to the COVID-19 pandemic and the lack of availability of faculty professors. Further, a decrease of the anxiety related to traditional suturing classes can be expected. We aim to create an innovative and sustainable method of teaching surgical skills to improve the efficiency and quality of surgical training in medical faculties. In the context of the COVID-19 pandemic, the need for such tools is imperative.

**Trial Registration:**

ClinicalTrials.gov NCT04425499; https://clinicaltrials.gov/ct2/show/NCT04425499

**International Registered Report Identifier (IRRID):**

PRR1-10.2196/21273

## Introduction

The necessity for medical students to develop proper suturing skills to achieve future excellence in surgery is sufficiently important to warrant interest in pursuing educational research with the goal of implementing systematic and effective methods of training [1]. Historically, the traditional methods by which medical students learn basic suturing skills have involved observation and trial during clerkship [2-4]. Currently, several issues pertaining to this practice in medicine have arisen due to the legal ethics involved [5] because both patients and students are at greater risk of injury. Without appropriate preparation, students are at higher risks of making mistakes [1,6]. Poor suturing technique and increased wound tension have been associated with substandard wound adhesion and increased scar formation [7]. The levels of comfort, confidence, and preparedness felt by medical students when performing these medical acts are also less developed [8], which may further adversely affect their performance when treating a real patient.

The academic learning objectives of medical students include several different suturing techniques. The most basic is the simple interrupted suture. A more advanced technique is the running subcuticular suture. All medical students are required to learn these surgical skills during their education, which is a source of anxiety and a challenge for many students.

With the traditional method, student learning depends on circumstances and the random presentation of cases [6]. The benefits of developing basic surgical skills before clerkship have been demonstrated. Early and prolonged exposure to and practice of suturing have been associated with an increased level of confidence relative to suturing a patient [2,4].

Acquiring this skill during preclinical years may increase the level of preparation of students, thereby increasing the professionalism and quality of the medical acts they perform on patients. However, the training in this technique during preclinical years has been limited due to time constraints on faculty staff as well as the cost of using these staff as trainers [9,10].

Several studies support peer-assisted training during preclinical years as a sustainable alternative [9,11-14]. These studies focus on basic surgical skills, such as uninterrupted stitches and knot-tying [15]. Although most studies demonstrate equivalence in acquisition of proficiency whether students are taught by peers or by faculty surgeons [12,13,16-18], it is unknown whether these results can be extrapolated to more advanced suturing skills, such as the vertical/horizontal mattress or running subcuticular sutures. In 2013, a study compared the execution of interrupted and subcuticular sutures among three groups: expert-trained, peer-trained, and self-taught. This study demonstrated that the execution by students in the peer-trained group was equivalent to that of the expert-trained students for both suture techniques [8]. Further studies pertaining to more advanced skills are warranted.

In the pursuit of developing sustainable alternatives in surgical education, several web-based tools have been gaining ground; tools that implement gamification elements are of particular interest, as they may further engage the student during their learning experience [19-21]. Several publications support gamified learning in health education due to its many unique advantages. Innovative platforms have the potential to provide effective, collaborative, inexpensive, and enjoyable opportunities to learn [19,21,22]. The Gamified Educational Network (GEN) is a web-based platform that was developed at Ontario Tech University and permits peer feedback through video assessment. Several educational tools have been developed and tested in surgical training [23-27]; however, the GEN platform enables students to learn a variety of skills without developing incorrect habits while practicing on their own. Students can upload videos of themselves performing a skill and share improvement tips with peers. Long-term retention of a surgical skill is optimal when the training is spread out over time rather than being provided in a single training session in one day [28]. Therefore, this platform offers superior psychomotor skill development by offering students the opportunity to expand the practice time of basic surgical skills beyond the classroom or clinical setting. In this way, students can practice skills and receive direct feedback at home.

During the present worldwide event of the COVID-19 pandemic, medical curricula have been suspended [29-31]. Therefore, offering students access to optimally efficient academic tools at home is of primordial importance [32]. The necessity for video instruction investigations is henceforth essential.

This study has four aims: (1) to evaluate students’ ability to identify the correct subcuticular suture method, comparing three web-based learning approaches of self-learning, peer-learning, and peer-learning with expert feedback; (2) to evaluate students’ ability to execute a proper running subcuticular suture, comparing three web-based learning approaches of self-learning, peer-learning, and peer-learning with expert feedback; (3) to determine the students’ acceptance of GEN to improve the platform as a learning tool; and (4) to develop an innovative web-based training and teaching method to better prepare students for simulation training and clerkship.

## Methods

### Study Design

This will be a prospective randomized controlled study with blinding of expert examiners. The study has been registered (ClinicalTrials.gov NCT04425499).

### Population

Participants are first-year medical students at Université de Montréal. We decided to target this population because these students do not yet have significant experience with suturing. We will invite the whole cohort (about 300 students) by email. 

### Eligibility and Ineligibility Criteria

#### Conditions for Eligibility

Students must be enrolled in their first year of medical school at Université de Montréal.

#### Conditions for Ineligibility

Students will be ineligible if they have returned after a leave, such as a sabbatical year, sick leave, or maternity leave; have already obtained a medical degree in another country; have studied medicine in another country; or are injured at the beginning of the study.

### Participant Recruitment

All students in their first year of medical school will receive an email inviting them to participate in the study. If they agree, they will be required to complete a consent form and provide a mailing address (Qualtrics XM).

All students enrolled in this study will be asked to participate using a personal computer. Students will be advised that participation will require their availability for one to three hours on three consecutive days. They will be advised not to study, practice, or view any external videos pertaining to suturing skills for the duration of the study.

When the students agree to participate, they will receive an email with the GEN platform link.

Students' previous experience with suturing will be surveyed, as well as the context of this experience (eg, workshop, former profession).

Upon creation of a new user account on GEN, students will be automatically randomized to one of the four groups and will only have access to the platform set for their study group.

### Randomized Groups and Study Intervention

**Figure 1:**
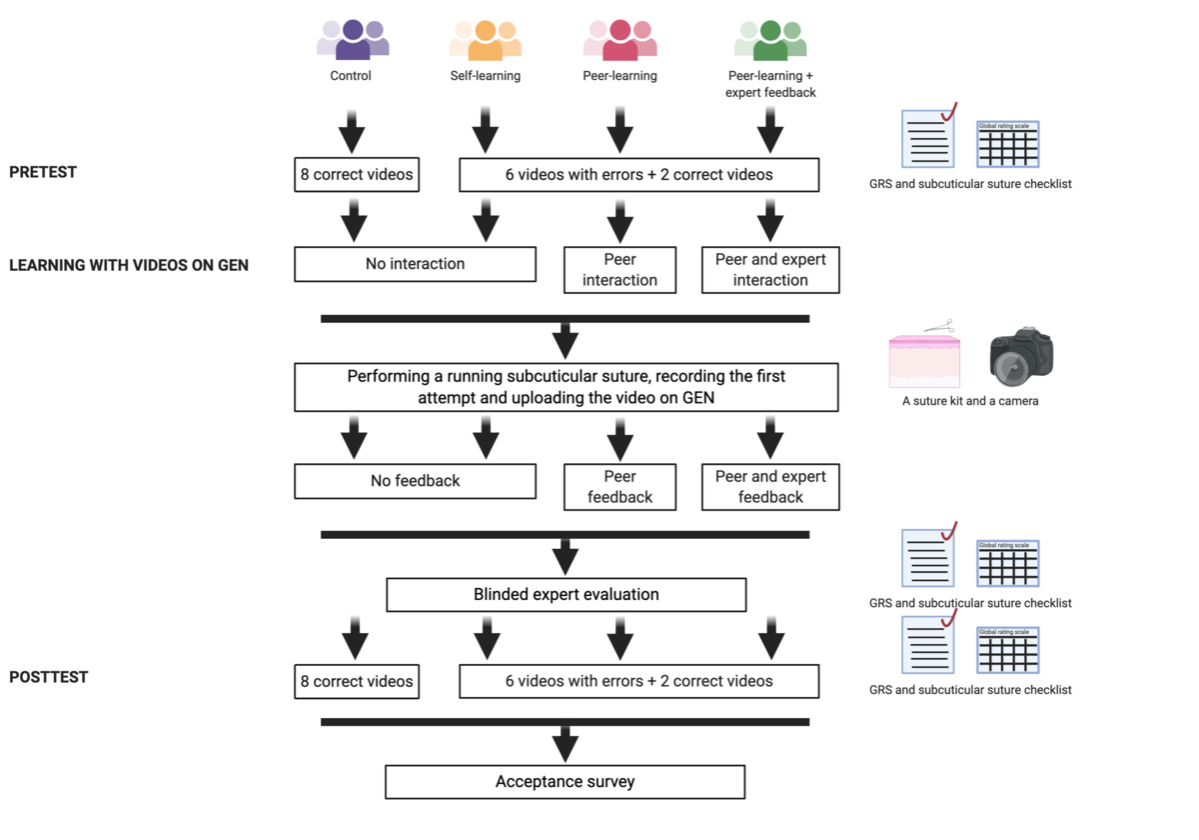
Study workflow. GEN: Gamified Educational Network; GRS: Global Rating Score.

Figure 1 depicts the study intervention.

#### Group 1: Control Group

On GEN, each student will individually view eight videos of an expert performing a running subcuticular suture correctly as a pretest. The Objective Structured Assessment of Technical Skills (OSATS) Global Rating Scale (GRS) (Table 1) and Subcuticular Suture Checklist (Textbox 1) will be made available beside each video, and students will be required to fill out these assessments for each video. For three days, students will have access to a distinct set of videos on GEN to learn running subcuticular sutures. Three days later, each student will perform a subcuticular suture on a suturing pad and record their first attempt. Then, students will undergo a posttest with the same eight videos shuffled in a different order. The order of the videos will be the same for all students.

The OSATS, developed by the University of Toronto, is an accurate and validated method of assessing surgical skills in an operating room setting [33,34]. It comprises a GRS (Table 1) in which certain skills are graded from 1 to 5 (ie, respect for tissue, time in motion, knowledge of instruments, instrument handling, suture skill, flow of operation, knowledge of procedures, and final product).

The checklist is a list of verifications specific to the technique of interest to ascertain whether every step of the procedure was executed (Textbox 1). The checklist was built and revised by a focus group led by the principal investigator, who is the director of the clerkship surgical rotation in the Faculty of Medicine of Université de Montréal. The focus group was composed of one medical student, one junior surgery resident, and one senior surgery resident.

**Table 1:** Global Rating Scores of the Objective Structured Assessment of Technical Skills [8,33,34]. The students will be asked to rate the candidate’s performance on a scale of 1 to 5, and the total score will be summed.

Skill	Potential scores for observed outcomes				
	1-2		3		4-5
Respect for tissue	Frequently used unnecessary force on tissues or caused damage by inappropriate instrument use		Careful handling of tissue, but occasional inadvertent damage		Consistently handled tissues appropriately with minimal damage
Time in motion	Many unnecessary moves		Efficient time and motion, but some unnecessary moves		Clear economy of movement and maximum efficiency
Instrument handling	Repeatedly makes tentative or awkward moves with instruments		Competent use of instruments, but occasionally awkward		Fluid movements
Suture skill	Awkward and unsure with poor knot tying and inability to maintain tension		Competent suturing with good knot placement and appropriate tension		Excellent suture control with correct suture placement and tension
Flow of operation	Frequently stopped operating, seemed unsure of next move		Demonstrated some forward planning and reasonable progression of procedure		Obviously planned operation
Knowledge of procedure	Inefficient knowledge of procedure. Looked unsure and hesitant		Knew all important steps of procedure		Demonstrated familiarity of all steps of procedure
Final product	Final product of unacceptable quality		Final product of average quality		Final product of superior quality
Overall performance	Very poor		Competent		Very good

**Subcuticular suture checklist. One point is given for each step performed correctly.**

**Preparation for the technique**

□      The student dons sterile gloves.

□      The student maintains sterility throughout the procedure.

**Appropriate use of suturing equipment**

□      Needles and tissue are always manipulated with equipment and not with hands.

□      The needle is properly held at a point two-thirds from its tip.

□      The needle holder is appropriately held with extended index and fourth fingers.

□      Forceps are properly held with the student’s nondominant hand.

**Execution of the running subcuticular suture**

□      Orientation of the needle upon insertion is appropriate (at 90º).

□      A deep dermal suture is performed on the extreme interior part of the wound.

□      The needle is properly exited between the deep dermal knot and the apex of the wound.

□      At least three knots are performed, with two loops around the needle holder for the first knot and 1 loop for the remaining knots.

□      The student alternates directions at 180º angles when tying the knot.

□      For each subcuticular insertion, the needle is properly inserted in the superficial layer of the dermis.

□      Each subcuticular insertion is appropriately spaced out.

□      Each bite is alternated on one side of the wound to the contralateral wound edge.

□      The surgical threads between each bite are parallel to each other.

□      A deep knot is performed at the other end of the wound.

□      Correct final knot technique is performed with the needle holder.

□      The final knot is exited lateral to the apex.

**Best practices**

□      Supination and pronation of wrists is performed.

□      The needle is protected at the end of the intervention.

□      The student can view the knot when it is cut and the scissors are held at a 45º angle.

**Final knot**

□      Proper tightness of the final knot is obtained.

□      The thread is cut at an appropriate length.

**Overall aesthetics**

□      Proper wound tension is displayed (no overlapping of tissues, no gaps).

Total: (  )

#### Group 2: Self-Learning

Each student will view eight videos individually and complete the GRS and Subcuticular Suture Checklist for each video as a pretest. Six videos will contain errors and two videos will not. The errors will be technical mistakes in the execution of a running subcuticular suture. Then, for three days, students will have access to a distinct set of videos on GEN to learn how to perform running subcuticular sutures. Three days later, each student will perform a subcuticular suture on a suturing pad and record their first attempt. Finally, students will perform a posttest with the same eight videos shuffled in a different order. The order of the videos will be the same for all students.

#### Group 3: Peer-Learning

Each student will view eight videos and complete the GRS and Subcuticular Suture Checklist for each video as a pretest. Six videos will contain errors and two videos will not. After this initial test, students will interact with other medical students in their group on the GEN platform anonymously for three days. We will display distinct videos on GEN. Comments will be allowed in an interactive format to encourage exchanges. Students will be required to participate in the discussion of at least two videos. Students will not be able to modify their answers on the initial test. Three days later, each student will perform a subcuticular suture on a suturing pad and record their first attempt. Students will interact on GEN and comment on the videos recorded by their peers. On the third day, the students in this group will perform a posttest with the same eight initial videos, but shuffled. The order of the videos will be the same for all students.

#### Group 4: Peer-Learning With Expert Feedback

The details for this group are mostly the same as for group 3; the only difference is that an expert will actively participate in the discussion by commenting on each video on GEN to enhance the students’ educational experience. Although the expert will be anonymous, the students will be able to identify them because they will use the name “expert.” The expert will answer any questions and comment on the discussion to guide the students.

### Study Intervention

In summary (Figure 1), the intervention consists of learning subcuticular sutures on the GEN platform with videos. In groups 1 and 2, there is no peer feedback. In group 3, the participants will learn the suturing skill *on GEN* by collaborating and interacting with each other. They will comment on the videos on GEN (ie, peer feedback). In group 4, the participants will receive peer and expert feedback.

Several gamification elements are integrated into the study to facilitate the interventions. First, the GRS and checklist are installed next to each video; therefore, the students can easily view the videos and properly check every step. Another gamification element in this system is similar to the entertainment, social networking, and news website Reddit, which supports peer-based assessment; peers will be able to comment on the videos, discuss them, and collaborate with each other.

GEN enables the application of a leaderboard. This social comparative feedback component provides learners with information regarding how well they are doing with respect to their peers. This comparative information is provided both individually and in a general context by showing the learner's position on a private individual leaderboard. The learners do not have access to the scores of their peers, avoiding comparisons that could be a detriment to motivation. Learners also obtain access to the number of points they received in each course section through an individual scoreboard.

GEN also provides division of the modules. When the participant has successfully completed one activity, the next activity is unlocked and displayed, much like a video game with successive levels. Moreover, there is a segmented progress bar that allows learners to track their progress in each course and also in each individual course component. As such, the tasks the students are required to complete are clearly displayed, similar to a map, with deadlines; this clearly guides the participants through the activities and completion of the learning material on GEN.

### Study Procedure

#### Objective 1: Evaluate Students’ Ability to Identify the Correct Subcuticular Suture Method

We will determine the best learning method in a virtual environment among the three main groups. We will compare groups 2, 3, and 4: self-learning, peer-learning, and peer learning with expert feedback. There will be no crossovers. Students will undergo a pretest and a posttest to assess the effect of the intervention on their ability to identify the correct way to perform subcuticular sutures. We will also use the control group to compare our intervention to the regular web-based learning method, in which students learn alone by watching different videos with no artificial errors inserted. We will analyze the students’ scoring of each video with the GRS and Subcuticular Suture Checklist.

#### Pretest and Posttest

All videos will show an expert performing a running subcuticular suture. The expert will be an experienced faculty surgeon licensed by the Royal College of Canada. The expert will only perform the suture without explanation; there will be no sound accompanying the videos. Among the eight videos displayed to students in groups 2, 3, and 4, six will contain technical errors. We will evaluate the students’ ability to identify the errors as they complete the GRS and the Subcuticular Suture Checklist. Participants in groups 2, 3, and 4 will all see the same videos in the same order. The OSATS GRS (Table 1) and Subcuticular Suture Checklist (Textbox 1) will be available beside each video in the pretest and posttest for every student in all four groups.

#### Errors

Each video with errors will include 1 to 3 errors from the list detailed below:

- The needle holder is held with the thumb and index finger instead of the thumb and fourth finger.
- The thumb is entirely inserted in the ring of the needle holder.
- The needle is not held at two-thirds in the jaw of the needle holder.
- Suturing is performed at an incorrect depth.
- Excessive force is used with the forceps.
- The needle is not inserted at a 90º angle.
- Sutures are at inappropriate distances.
- Extra unnecessary steps are performed.
- The knot is not sufficiently tight.
- Supination and pronation are not properly performed.
- The thread is cut at an inappropriate length after tying the knot.
- The thread is cut without seeing the knot.

#### Objective 2: Evaluate Students’ Ability to Execute a Proper Running Subcuticular Suture

We will determine the best learning method in a virtual environment among the three main groups. We will compare groups 2, 3, and 4: self-learning, peer-learning, and peer learning with expert feedback. There will be no crossovers. Students will receive a suture kit and perform a running subcuticular suture on a synthetic suturing pad. They will record their first attempt and upload it on GEN. One expert will evaluate the students’ performance in the uploaded videos using the GRS and the Subcuticular Suture Checklist. The expert will be a faculty surgeon licensed by the Royal College of Canada. The results will not be published on GEN. The expert will be blinded to the study groups. We will require students to set up their camera field identical to that in the expert videos they will watch on GEN. This will prevent variations in ratings that could occur due to differences in the participants’ setups. More specifically, the learners will be instructed to adjust the field of view of the camera as follows:

- Camera location: The camera must face the front and be positioned at a height of 30-50 cm.
- Field of view: The entire simulator must be visible. Instruments and hands (up to the elbows) must be visible at all times. Students must wear gloves and ensure that there are no objects within the camera’s field of view that can be used to identify them.
- Resolution and frequency: The camera should be set to 1080p resolution at 24 frames per second.
- Format: The students are asked to ensure that the videos are captured in mp4 or mov file formats.

We will also use the control group to compare our intervention to the regular web-based learning method in which students learn alone by watching different videos with no artificial errors inserted.

The package of the suture kit will contain an instruction to only open the box while recording the video in which the first attempt at suturing is performed. The student must demonstrate that the package is opened only while recording the video to ensure that they do not practice beforehand. Only the suture pad and the student’s forearms will be shown in the videos to maintain anonymity. Students will not have access to the uploaded videos of the other students.

#### Objective 3: Determine the Students’ Acceptance of GEN to Improve the Platform as a Learning Tool

At the end of the study, the students will fill out a survey [35] on their experience with the GEN platform as a learning tool (Textbox 2). This survey is anonymous.

**Survey to evaluate the students’ acceptance of GEN.**

1. In this study, there were four experimental groups, and you were enrolled randomly to one of these four groups. Based on your experiences and interactions within GEN, which group do you think you were enrolled in?

     a. Group 1. Control

     b. Group 2. Self-learning

     c. Group 3. Peer-learning

     d. Group 4. Peer-learning with expert feedback

2. Please rate the level of agreement with the following statements, where 1 means “strongly disagree,” 2 means “disagree,” 3 means “neutral,” 4 means “agree,” and 5 means “strongly agree.”

     a. I found GEN to be a user-friendly platform.

     b. I found GEN to be a useful platform for improving my suturing skills.

     c. I would recommend GEN to other medical students.

3. Based on your recent experience, please provide a very brief description of features of GEN that you think we should:

     a. Stop (one feature that we should eliminate)

     b. Start (one feature that we should add)

     c. Change (one feature that works but could be improved upon)

     d. Continue (one feature that we should retain and not change)

Questions 4 to 12 are derived from the System Usability Scale [35]. The scale ranges from 1 to 5.

4. Please rate the level of agreement with the following statements, where 1 means “strongly disagree,” 2 means “disagree,” 3 means “neutral,” 4 means “agree,” and 5 means “strongly agree.”

     a. I would like to use GEN frequently.

     b. I found GEN unnecessarily complex.

     c. I thought GEN was easy to use.

     d. I would need the support of a technical person to be able to use GEN.

     e. I found the various functions in GEN were well integrated.

     f. I thought there was too much inconsistency in GEN.

     g. I would imagine that most people would learn to use GEN very quickly.

     h. I found GEN very cumbersome (awkward) to use.

     i. I felt very confident using GEN.

     j. I needed to learn a lot of things before I could get going with GEN.

5. During the study, did you access any external resources to help you with the material on GEN?

     a. Yes (if yes, please answer question 6)

     b. No (If no, you are finished with the survey))

6. If you did indeed access external material, please list these resources below.

#### Objective 4: Develop an Innovative Web-Based Training and Teaching Method to Better Prepare Students for Simulation Training and Clerkship

The GEN platform was built by the research group of Dr Adam Dubrowski at Ontario Tech University. In collaboration with this research group, the platform will be further developed for our study. Each study group will have its own sector on the platform. Therefore, students from one group will not have access to the comments and web-based activity of the other groups. The pretest and the posttest performed in objective 1 will be on GEN, as will the videos uploaded by students in objective 2.

### Materials

Students will require a computer with internet access and a camera.

The pretest and posttest videos will be recorded beforehand using synthetic suture pads and the following instruments: a needle holder, a sterile suture, forceps, and latex gloves.

All students will receive a suturing kit by mail composed of a synthetic suture pad, a needle holder, a sterile suture (3-0 Vicryl), forceps, and latex gloves.

### Sample Size

The experiment is designed so that each of the four groups will have the same sample size. We calculated the sample size based on our primary objective considering a balanced one-way analysis of variance (ANOVA) model. We used the GRS for the calculation.

In the study by Denadai et al [8], subcuticular sutures were assessed using the GRS among three intervention groups. The difference between the lowest mean and the highest mean was 12.25. In that study, we approximated the mean standard deviation within groups to be 1.45. We chose to set the effect size at 0.8 because we do not know the overall standard deviation for all participants. We set the power to 80% and the significance level to 0.05.

Using RStudio, we obtained a sample size of 13 participants per group and a total sample size of 52. We will include 15 participants in each group (total N=60) to have sufficient statistical power. We will invite the whole cohort of first-year medical students (n=300) and enroll the first 60 students who reply favorably.

### Ethics

This study will be conducted following the Declaration of Helsinki on human research. The protocol has been approved by our institutional review board (CERSES 20-068-D). Any amendment to the protocol must be approved by this committee. A consent form will be sent to the students. Participation in this study is voluntary. Students will not be exposed to any risks apart from those inherent in the manipulation of sterile needles. This small risk will be mentioned in the consent form and is considered to be low compared to the benefits of the study.

Students’ anonymity will be maintained on the GEN platform throughout the study.

## Results

### Data Collection

All data will be anonymous, and files will be secured with a password. In objective 1, the results of the GRS and Subcuticular Suture Checklist for all participants will be collected through GEN. In objective 2, the expert evaluating the videos will compile the data in one file that will not be published on GEN. Responses from the acceptance survey will be collected using Qualtrics XM.

### Statistical Analysis Plan

First, we will perform descriptive statistical analyses to assess the distributions of the variables of interest and determine the appropriate tests to use as well as the need for a potential transformation. Continuous variables with an asymmetric distribution will be described using median (IQR). Categorical variables will be described with proportions. The remaining variables will be described with mean (SD).

In the following paragraphs, we describe our statistical plan assuming a Gaussian distribution.

Objective 1: We will perform balanced one-way ANOVA to compare mean GRS and Subcuticular Suture Checklist scores among all four groups. First, distinct analyses of the pretest and the posttest scores will be performed. Then, the mean difference between the pretest and the posttest scores will be compared using paired *t* tests if the difference between paired values is consistent.

Objective 2: We will perform balanced one-way ANOVA to compare the performance of students in executing a running subcuticular suture as assessed by the GRS and the Subcuticular Suture Checklist.

Objective 3: We will perform descriptive statistical analyses and correlate the study groups with the Likert scales using the Kruskal-Wallis equality-of-population rank test.

Analysis will be performed in RStudio version 1.2.5033 (RStudio Team) [36] and GraphPad Prism version 8.0.0 for Mac OS X (GraphPad Software) [37]. For each analysis, confidence intervals will be calculated for a 95% confidence level. Statistical hypothesis testing will be performed with an alpha threshold of .05.

## Discussion

### Significance

In this study, we will compare alternative learning methods of an advanced surgical skill. The role of distant peer-learning with or without the involvement of an expert will help reinvent surgical skill teaching. Also, the interactive aspect of the GEN platform will improve the students’ learning experience while reducing their anxiety. Students will be able to share tips and to progress with their team from home. Students participating in this study will have the opportunity to gain suturing knowledge and to help improve surgical education.

This study has potential to create a sustainable educational method for the acquisition of advanced suturing techniques. We will develop a web-based training and teaching method to better prepare students for simulation training and clerkship, especially when there is no access to traditional training methods in the context of the COVID-19 pandemic. Another important advantage is the alleviation of the burden carried by surgical professors, who have a considerable number of responsibilities.

Our goal is to eventually allow students to upload their own videos on GEN and to interact with other students and with an academic surgeon. Learning on GEN with teammates and expert feedback is promising for students, as they can receive accurate feedback with lower risks of acquiring incorrect habits.

### Study Limitations

The suturing skills of the students before this study are unknown. However, this factor is controlled because all students will be randomized. Only students in their first year of medical school are eligible to participate the study. At this stage of their education, they have not yet received any training in surgical or suturing skills. However, students who are more interested in surgery and with more experience are more likely to participate. This can lead to small effect differences between groups.

A possible limitation is that students may access external material such as other videos on the web or through practicing on their own. However, we will require students to self-report external material they have used or additional practicing they performed. These questions will be asked in the poststudy survey. Because the students will be in their first year of medical school, they will have poor suturing knowledge. Further, the students will only have three days to complete the study and learn a practical skill, which limits the time they can spend on external platforms. Therefore, we do not believe that accessing external material will have a considerable impact on the outcomes.

Learning and practicing for three days at home is not directly comparable to the traditional method in which a single surgeon teaches suturing skills for one to three hours at a university. In this study, we aim to compare different web-based learning methods for a specific skill. Learning on GEN cannot replace the traditional method. Rather, we believe that it represents a potential adjunct and an optimal option in the context of the COVID-19 pandemic.

A vast array of gamification elements have been previously published [19-22]. However, we decided to limit the number of gamification elements used in this study and to focus on our primary objective of evaluating procedural knowledge. Further, limiting the number of gamification elements helps simplify distance learning of an advanced suturing skill for novice learners who are not familiar with that skill.

Other groups have used constructs to study distinct outcomes, such as students' fear of making mistakes, perceived knowledge of how to behave, perceived errors committed, and attitudes [38]. These subjective variables would be interesting to assess in subsequent studies as well in the surgical field. We believe that GEN could decrease students' anxiety when learning advanced suturing techniques compared to the traditional method [39]. In our study, we will assess user experience on GEN using the System Usability Scale.

## Abbreviations

ANOVA: analysis of variance
GEN: Gamified Educational Network
GRS: Global Rating Scale
OSATS: Objective Structured Assessment of Technical Skills
